# A latent profile analysis of resourcefulness among patients with colorectal cancer and intestinal ostomy

**DOI:** 10.3389/fpsyg.2026.1906523

**Published:** 2026-08-19

**Authors:** Xiaocui Zou, Li Xiao, Ling Hu, WenQing Guo, AiXin Zhou, XiaoJuan Yang, Qing Wen, Hua Zhang, Hong Wu

**Affiliations:** 1 Department of Hepatobiliary and Pancreatic Surgery, Sichuan Provincial People's Hospital, University of Electronic Science and Technology of China, Chengdu, Sichuan, China; 2 Department of Geriatric Cardiology, Sichuan Provincial People's Hospital, University of Electronic Science and Technology of China, Chengdu, Sichuan, China; 3 Department of Gastrointestinal Surgery, The First Affiliated Hospital of Chongqing Medical University, Chongqing, China; 4 Department of Nursing Department, The First Affiliated Hospital of Chongqing Medical University, Chongqing, China; 5 Department of Urology Surgery, Sichuan Provincial People's Hospital, University of Electronic Science and Technology of China, Chengdu, Sichuan, China; 6 Department of Nursing Department, Sichuan Provincial People's Hospital, University of Electronic Science and Technology of China, Chengdu, Sichuan, China; 7 Department of Gastrointestinal Surgery, Sichuan Provincial People's Hospital, University of Electronic Science and Technology of China, Chengdu, Sichuan, China

**Keywords:** colorectal cancer, enterostomy, influence factor, latent profile analysis, resourcefulness

## Abstract

**Background:**

Patients with colorectal cancer and an intestinal ostomy face significant psychological challenges after surgery. Resourcefulness is crucial for psychological adaptation, but its heterogeneity and associations with stress, acceptance, and stigma remain unclear.

**Objective:**

This study aimed to identify distinct resourcefulness profiles among these patients and examine their associations with sociodemographic factors, perceived stress, stoma acceptance, and stigma.

**Methods:**

A convenience sampling method was adopted to recruit 479 patients with colorectal cancer and intestinal ostomy from the gastrointestinal surgery departments of Class A tertiary–level hospitals in Chengdu and Chongqing, China, between February and December 2025. Data were collected using the Resourcefulness Scale (RS), the Perceived Stress Scale (PSS), the Stoma Acceptance Questionnaire (SAQ), and the Social Impact Scale (SIS). Latent profile analysis was performed to identify subgroups, and multinomial logistic regression and ANOVA were used to examine associated factors and psychosocial differences.

**Results:**

A 4-class model was optimal: Low Resourcefulness (C1, 28.60%), Medium Resourcefulness–Intimate-Reliant (C2, 17.33%), High Resourcefulness–Selectively Help–Seeking (C3, 36.12%), and High Resourcefulness (C4, 17.95%). Married status, higher education, higher income (≥5,000 CNY), and participation in ostomy support meetings were key distinguishing factors between the high-resourcefulness profile (C4) and the low-resourcefulness profile (C1), with the proportions of patients possessing these characteristics increasing progressively from C1 to C4. A clear gradient emerged: from C1 to C4, perceived stress and stigma decreased, while stoma acceptance increased (all *p* < 0.001).

**Conclusions:**

Resourcefulness in this population is heterogeneous. Marital status, education, income, and professional support engagement are key factors influencing profile membership.

**Implications for Oncology Nursing Practice:**

Nurses should assess patients' resourcefulness to tailor support. Actively encouraging participation in structured ostomy support meetings is crucial. Interventions should be profile-specific: providing comprehensive resource reinforcement for C1, enhancing support networks for C2, and offering autonomy-respecting guidance for C3.

**Trial registration:**

Chinese Clinical Trial Registry; http://www.chictr.org.cn/;ChiCTR2500095532.

## Background

Colorectal cancer (CRC) is one of the most common malignant tumors of the digestive tract globally, with rising incidence and mortality rates, posing a significant public health challenge (Nagtegaal et al., 2020; Sung et al., 2021). According to 2022 cancer statistics in China, there were approximately 517,000 new cases of colorectal cancer, accounting for 10.7% of all new cancer cases, ranking second in the spectrum of cancer incidence (Han et al., 2024). The number of deaths reached about 240,000, representing 9.3% of total cancer-related deaths, ranking fourth nationwide. In southwestern China (Lin et al., 2024), the burden of colorectal cancer is particularly pronounced, with standardized mortality rates among the highest in the country, indicating an urgent need for focused prevention and control efforts in this region.

Currently, surgical resection is the primary treatment for colorectal cancer. Enterostomy, as a common surgical intervention, can effectively save lives but also brings significant physiological and psychological challenges (National Health Commission of the People's Republic of China, 2020). It is estimated that approximately 100,000 patients in China undergo enterostomy surgery annually, with a growing trend (Meng and Xu, 2021). In the early postoperative period, patients often experience psychological distress—including increased perceived stress (Jin et al., 2019), decreased stoma acceptance (Li et al., 2018), and heightened stigma—due to changes in bowel habits, complex stoma care, and body image disturbance (Du and Li, 2022). These reactions severely impact patients' physical and mental health as well as their quality of life.

With the deepening application of positive psychology in the medical field, researchers have gradually shifted their focus from pathological issues to exploring patients' positive psychological resources (Seligman, 2019). Resourcefulness, defined as an individual's comprehensive ability to mobilize internal and external resources to cope with challenges, plays a key role in the psychological adaptation of this population (Huang et al., 2018). Understanding the relationship between resourcefulness and specific psychosocial variables is crucial for building an effective support system.

First, resourcefulness is associated with patients' stress regulation processes (Bekhet, 2014). A colorectal cancer diagnosis and enterostomy surgery constitute major stressors, often plunging patients into states of tension and loss of control. Research shows that individuals with higher levels of resourcefulness perceive significantly lower levels of stress (Li, J. et al., 2025). Studies by Guo et al. (2019) indicate that resourcefulness is related to a weaker association between perceived stress and negative emotions. For enterostomy patients, higher resourcefulness has been linked to better utilization of internal self-regulation strategies (such as positive thinking and problem planning) and external social support resources, which may contribute to the management of physical and psychological impacts of the disease and its treatment and are associated with lower stress levels (Liao et al., 2024).

Second, resourcefulness serves as an important psychological resource for resisting stigma. Due to changes in health status and bodily functions, enterostomy patients are prone to feelings of being labeled and socially excluded. This stigma is closely linked to their level of resourcefulness (Danielsen et al., 2013). Research by Zhang et al. (2022) clearly demonstrates a significant negative correlation between patients' resourcefulness and their level of stigma. Patients with higher resourcefulness tend to understand their condition through positive cognitive reappraisal and actively seek emotional support and practical assistance from trusted individuals or professional channels, rather than passively succumbing to self-deprecation and social isolation (Wang et al., 2015), a tendency that is associated with more effective mitigation of the impact of stigma on their social functioning and mental health.

Ultimately, resourcefulness is significantly associated with patients' acceptance and adaptation to the stoma (Wang et al., 2021). Psychological acceptance of the stoma is a cornerstone for patients' return to normal life. The personal competencies (such as self-control and problem-solving) and social competencies (such as effective help-seeking) encompassed by resourcefulness theory jointly facilitate this adaptation process (Muzii et al., 2024). Whether patients can view stoma care positively, experience autonomy in self-care, and evaluate and utilize available support are all closely related to their level of resourcefulness (Szpilewska et al., 2018). Higher resourcefulness is associated with a transition from passive endurance to active management, and correlates with a stronger sense of control over life as well as greater cognitive, emotional, and behavioral acceptance of the stoma.

However, existing research on the resourcefulness of patients in the early postoperative period after enterostomy for colorectal cancer remains insufficient, particularly lacking comprehensive investigations that integrate perceived stress, stoma acceptance, and stigma. Moreover, most studies treat patients as a homogeneous group, overlooking the potential classification of their psychological characteristics, which may constrain the targeted effectiveness of interventions. Therefore, this study aims to identify homogeneous groups of colorectal cancer enterostomy patients based on their latent resourcefulness characteristics, examine the sociodemographic correlates of these characteristics, and explore the relationships between these latent characteristics and perceived stress, stoma acceptance, and stigma.

## Methods

### Participants

**Figure 1:**
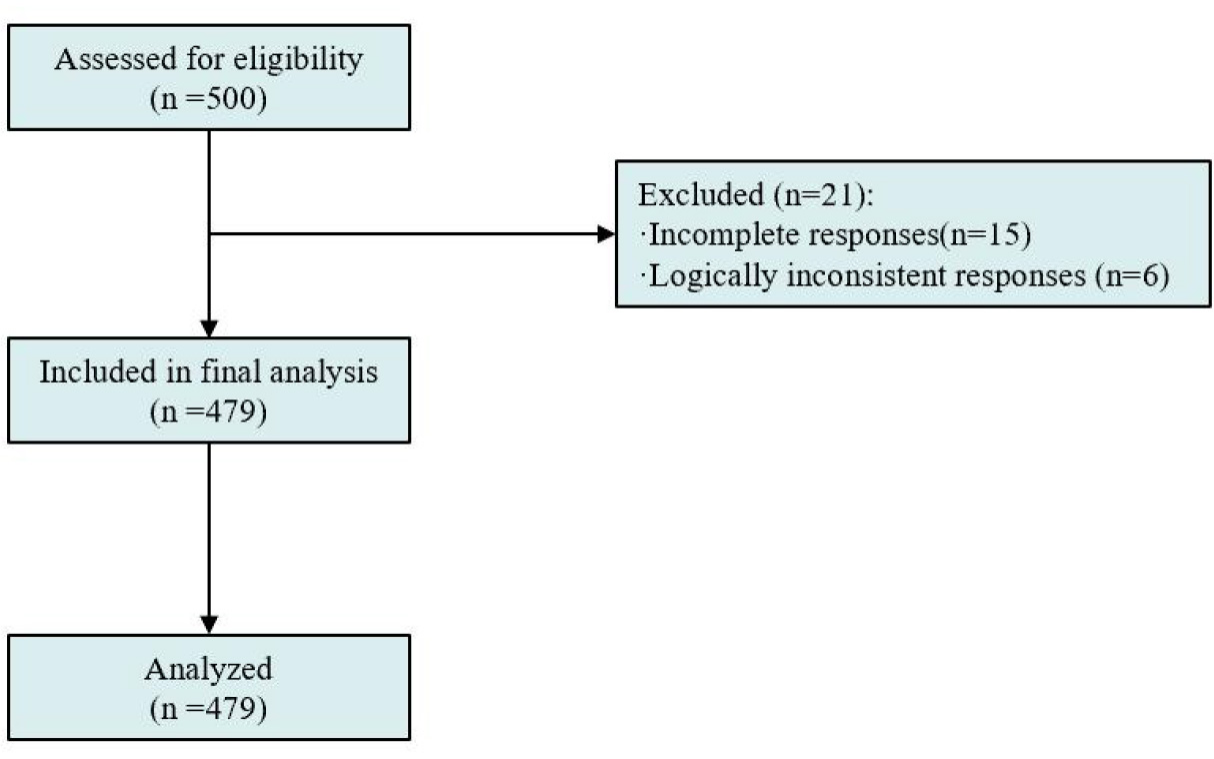
Participant flow diagram. The use of convenience sampling from two tertiary hospitals limits generalizability and should be emphasized in the discussion.

An exploratory cross-sectional study was conducted in the Sichuan-Chongqing region of China between February and December 2025. Participants were recruited via convenience sampling from the gastrointestinal surgery departments of two Class A tertiary hospitals: Sichuan Provincial People's Hospital (Chengdu; *n* = 200) and The First Affiliated Hospital of Chongqing Medical University (Chongqing; *n* = 300). All participants were assessed within 30 days following their initial ostomy surgery. Sample size determination for latent profile analysis followed contemporary methodological recommendations. Monte Carlo simulation studies indicate that adequate sample sizes for LPA depend on multiple factors, including the number of indicators, class separation, class proportions, and model complexity (Nylund et al., 2007; Tein et al., 2013). For models with 2–5 classes and moderate class separation, sample sizes of approximately 200–500 participants generally provide sufficient statistical power and stable parameter estimates (Wurpts and Geiser, 2014). Given our use of 28 indicators (the Resourcefulness Scale items), expected class proportions, and the need to detect smaller classes, we aimed for a sample size at the upper end of this range. Additionally, simulation studies have shown that entropy values and parameter recovery improve with sample sizes exceeding 300, particularly when class separation is moderate (Kim, 2014; Muthén and Muthén, 2000). Based on these considerations and accounting for a 5% anticipated non-response rate based on our previous multicenter studies in this population, we enrolled 500 participants. Of the 500 distributed questionnaires, 21 were excluded from the final analysis. The reasons for exclusion were incomplete responses with >10% missing items (*n* = 15) and logically inconsistent responses (e.g., contradictory answers to reverse-coded items, *n* = 6). The remaining 479 valid questionnaires constituted the final analytic sample, which was complete with no missing data points (Figure 1). The final analytic sample of 479 provided sufficient statistical power for the LPA, as evidenced by the robust model fit indices, clear class separation (entropy = 0.966), and well-balanced class proportions (smallest class = 17.33%). The inclusion criteria were (a) meeting the diagnostic criteria for colorectal cancer with a pathologically confirmed diagnosis (Li et al., 2024); (b) undergoing initial ostomy surgery; (c) being aged 18 years or over; and (d) voluntarily participating with signed informed consent. Exclusion criteria: (a) severe psychiatric disorders or cognitive impairment, assessed through medical record review, physician judgment, and the participant's ability to complete the questionnaire independently. (b) had concurrent primary tumors in other sites. (c) were receiving concurrent radiotherapy or chemotherapy at the time of assessment.

### Procedures

Three nurses with research backgrounds and over 5 years' experience in clinical practice were selected from within the department to serve as investigators. The project leader provided standardized training to all three investigators. Before initiating the survey, the investigators were required to explain the study thoroughly to participants, obtain their consent, and have them sign informed consent forms. They then used standardized guidance language to explain the study's purpose, content, significance, and key considerations for completing the questionnaire. While participants were completing the questionnaire, the investigators had to promptly address any questions while avoiding leading language. Once the questionnaires had been completed, the investigators collected them on-site and verified their completeness and the logical consistency of the items. Participants should then be asked promptly to supplement or correct any errors, omissions, or inconsistencies. This study has been approved by the hospital ethics committee (Approval No. 2022-409) and adheres to the principles outlined in the Declaration of Helsinki.

### Measures

#### Socio–demographic questionnaire

Our team independently designed the sociodemographic survey questionnaire based on a systematic review of relevant domestic and international literature. It includes questions on gender, age, educational attainment, marital status, per capita monthly household income, primary residence, occupation, type of health insurance, and participation in ostomy support meetings (defined as having attended at least one hospital-organized ostomy peer support group session since surgery). These meetings were facilitated by stoma care nurses and included both educational components and peer experience sharing. Sessions were held weekly, each lasting approximately 40 min. For the purpose of this study, attendance was recorded as a binary variable (yes/no), indicating whether the patient had participated in at least one such meeting prior to completing the questionnaire, any underlying medical conditions, surgical approach, type of stoma, and any stoma complications. Patients complete the questionnaire independently, and it is verified against electronic medical records.

#### Resourcefulness Scale (RS)

The RS, developed by (Zauszniewski et al., 2006), was translated into Chinese by (Wang et al., 2015). It assesses an individual's level of resourcefulness. It comprises two dimensions — personal and social resourcefulness — with a total of 28 items. Sixteen of these items measure personal resourcefulness, while the remaining 12 items assess social resourcefulness. The scale uses a 6-point Likert scale, with response options ranging from Level 1 (“Strongly Disagree”) to Level 6 (“Strongly Agree”), which correspond to scores from 0 to 5. Total scores range from 0 to 140, with higher scores indicating greater resourcefulness. The scale's Cronbach's α is 0.85 (Zauszniewski et al., 2006). In this study, the Cronbach's α for the scale was 0.797.

#### Perceived Stress Scale (PSS)

The PSS, developed by (Cohen et al., 1983), is designed to assess how stressful individuals perceive their lives to be over the preceding month. This study employed the Chinese version of the scale, which was translated and revised by (Yang and Huang, 2003). The scale comprises 14 items across two dimensions: tension and loss of control. Responses are recorded on a 5-point Likert scale ranging from 0 (“never”) to 4 (“very often”). Items 4, 5, 6, 7, 9, 10, and 13 are positively worded but are reverse-scored. The total score ranges from 0 to 56; a higher score indicates a greater level of perceived stress. The reported Cronbach's α for the original scale is 0.78 (Yang and Huang, 2003). In the present study, the Cronbach's α was 0.894.

#### Stoma Acceptance Questionnaire (SAQ)

The SAQ, developed by Bagnasco et al. (2017) was used to assess patients' acceptance of their intestinal ostomy. The questionnaire comprises 12 items across three dimensions: four items assess importance, five assess autonomy and acceptance, and three assess perceived support. It employs a 4-point Likert scale. For the importance dimension, responses range from one (“not at all important”) to four (“very important”). For autonomy and acceptance, they range from one (“not at all good”) to four (“very good”). For perceived support, responses range from one (“strongly disagree”) to four (“strongly agree”). The total score ranges from 12 to 48 points, with higher scores indicating greater acceptance of the stoma. The scale has demonstrated good reliability in prior studies involving ostomy patients, with a reported Cronbach's α of 0.83 (Hu et al., 2020). In this study, the Cronbach's α for this scale was 0.966.

#### Social Impact Scale (SIS)

The SIS was developed by Fife and Wright (2000). to measure the level of stigma experienced by patients with chronic diseases, such as cancer. This study used the Chinese version of the SIS, which was translated by (Pan et al., 2007). The scale comprises four dimensions—social exclusion, financial insecurity, internalized shame, and social isolation—and contains a total of 24 items. It uses a 4-point Likert scale, with four representing “strongly agree” and one representing “strongly disagree.” All items are reverse-scored, yielding a total score ranging from 24 to 96 points. Higher scores indicate greater levels of stigmatization. The Cronbach's α for the scale was 0.92 (Pan et al., 2007). In this study, it was 0.974.

### Data analysis

Data were analyzed using Mplus version 8.3 and IBM SPSS Statistics version 23.0. The analysis consisted of three main steps. As the data were collected via self-report questionnaires at a single time point, Harman's single-factor test was performed to assess potential common method bias. An exploratory factor analysis was conducted on all items from the RS, PSS, SAQ, and SIS. This analysis revealed that the first unrotated factor accounted for only 31.33% of the total variance, which is well below the critical threshold of 40%. This suggests that common method bias was not a significant issue in this study.

First, descriptive statistics were performed using SPSS 23.0. Continuous variables were expressed as mean ± standard deviation, and categorical variables were described using frequencies and percentages.

Second, Latent Profile Analysis (LPA) was conducted using Mplus 8.3 to identify subgroups based on patients' resourcefulness. The 28 items of the Resourcefulness Scale served as the indicator variables. All indicators were treated as continuous variables and analyzed using the robust maximum likelihood (MLR) estimator, which provides standard errors and fit statistics robust to non-normality. To ensure global solution optimisation and avoid convergence to local maxima, we used an extensive random-start procedure. The analysis specified 500 random starting values for the initial stage, with the 100 best solutions retained for final-stage optimisation (Mplus command: STARTS = 500 100). Each random start ran for up to 100 iterations (STITERATIONS = 100). For the Bootstrap Likelihood Ratio Test (BLRT) and Lo-Mendell-Rubin adjusted likelihood ratio test (LMR), we specified 500 random starts with 250 final-stage optimisations (LRTSTARTS = 0 0 500 250) to ensure stable test results (Nylund-Gibson et al., 2023). The average posterior probabilities for each class were ≥ 0.95 (C1: 0.990, C2: 0.988, C3: 0.979, C4: 0.968), and the entropy was 0.966, both indicating excellent classification accuracy. The following fit indices were used to determine the optimal model: the Akaike Information Criterion (AIC), Bayesian Information Criterion (BIC), and adjusted Bayesian Information Criterion (aBIC), with lower values indicating better fit. The Lo-Mendell-Rubin adjusted likelihood ratio test (LMR) and the Bootstrap Likelihood Ratio Test (BLRT) were also used. Significant *p*-values (*p* < 0.05) from these tests suggest the k-class model is superior to the (k-1)-class model. Entropy was examined to assess classification accuracy; values closer to one (≥0.8 indicates accuracy ≥ 90%) reflect clearer profile delineation. The model selected balanced fit, parsimony, and interpretability (Adhikari et al., 2021).

Third, after identifying the optimal latent profiles, statistical analyses were performed using SPSS 23.0. Chi-square tests and one-way analyses of variance (ANOVA) were used to examine differences among profiles in categorical (e.g., demographic characteristics) and continuous (e.g., perceived stress, stoma acceptance, stigma) variables, respectively. Before conducting the one-way ANOVAs comparing psychosocial outcomes across the four profiles, we assessed the assumptions of normality and homogeneity of variance. Normality was examined using the Shapiro–Wilk test, and homogeneity of variance was assessed using Levene's test. For variables where the homogeneity assumption was violated, we employed Welch's ANOVA, which is robust to unequal variances and group sizes. *Post-hoc* comparisons were conducted using the Bonferroni test, which does not assume equal variances. Effect sizes are reported as η^2^ with 95% confidence intervals. Given the exploratory nature of these comparisons, we did not adjust for multiple testing to avoid Type II errors while acknowledging this limitation (Bender and Lange, 2001). Finally, variables showing significant differences in univariate analyses were included as independent variables in a multivariable multinomial logistic regression analysis. Profile membership was the dependent variable, using the “Low Resourcefulness” profile as the reference category. This approach identified factors associated with profile membership. Model fit was assessed using the-2 log-likelihood, Cox and Snell *R*^2^, and Nagelkerke *R*^2^. Multicollinearity was examined using variance inflation factors (VIF), with VIF > 10 indicating problematic collinearity (Hair et al., 2010). We assessed for sparse-cell issues by examining expected frequencies in each covariate-class combination; all exceeded 5, indicating adequate data for stable estimation. A two-sided *p*-value < 0.05 was considered statistically significant for all tests.

## Results

### Participant characteristics

A total of 500 questionnaires were distributed for this study, with 479 valid responses ultimately returned, yielding a valid response rate of 95.80%. The mean age was 63.961 ± 1.55 years (range, 19–95). The sample comprised 319 men (66.6%) and 160 women (33.4%), with 292 patients (61.0%) over 60 years old. Nearly half (50.7%, *n* = 243) had an education level of junior high school or below. Most were married (65.8%, *n* = 315), lived in urban areas (64.3%, *n* = 308), and were employed (55.3%, *n* = 265). The most common monthly household income per capita was 3,000–4,999 CNY (39.3%, *n* = 188). In terms of medical insurance, 36.3% (*n* = 174) were covered by the New Rural Cooperative Medical Scheme and 32.8% (*n* = 157) by Urban-Rural Resident Basic Medical Insurance. Clinically, the majority underwent laparoscopic surgery (88.3%, *n* = 423). A total of 350 patients (73.1%) had a temporary stoma. Although 198 patients (41.3%) had attended ostomy support meetings. Underlying medical conditions were reported by 205 patients (42.8%). Stoma complications were present in 45 patients (9.4%). Mean scores on the psychosocial scales were as follows: PSS total score: 28.241 ± 1.83; SAQ total score: 48.671 ± 5.65; SIS total score: 63.111 ± 9.29. The demographic characteristics of the participants are shown in Table 1.

**Table 1:** Demographic and clinical characteristics of participants (*N* = 479).

Variable	Category	*n* (%)
Gender	Male	319 (66.6)
	Female	160 (33.4)
Age (years)	≤ 60	187 (39.0)
	>60	292 (61.0)
Educational attainment	Junior high school or below	243 (50.7)
	High school/Technical secondary school	117 (24.4)
	College or above	119 (24.9)
Marital status	Married	315 (65.8)
	Unmarried/Divorced/Widowed	164 (34.2)
Per capita monthly	< 3,000	189 (39.5)
household	3,000–4,999	188 (39.2)
income (CNY)	≥5,000	102 (21.3)
Primary residence	Rural	171 (35.7)
	Urban	308 (64.3)
Occupation	Employed	265 (55.3)
	Unemployed	84 (17.6)
	Retired	130 (27.1)
Type of health insurance	Out-of-pocket	39 (8.1)
	New Rural Cooperative Medical Scheme	174 (36.3)
	Urban-Rural Resident Basic Medical Insurance	157 (32.8)
	Urban Employee Basic Medical Insurance	109 (22.8)
Ostomy support	No	281 (58.7)
meetings	Yes	198 (41.3)
Any underlying medical	No	274 (57.2)
conditions	Yes	205 (42.8)
Surgical approach	Open surgery	56 (11.7)
	Laparoscopic surgery	423 (88.3)
Type of stoma	Temporary	350 (73.1)
	Permanent	129 (26.9)
Stoma complications	No	434 (90.6)
	Yes	45 (9.4)
PSS Total Score		28.24 ± 11.83
SAQ Total Score		48.67 ± 15.65
SIS Total Score		63.11 ± 19.29

Data are presented as *n* (%) for categorical variables and Mean ± Standard Deviation (M ± SD) for continuous variables.

PSS, perceived stress scale; SAQ, stoma acceptance questionnaire; SIS, social impact scale.

### Latent profile analysis

**Figure 2:**
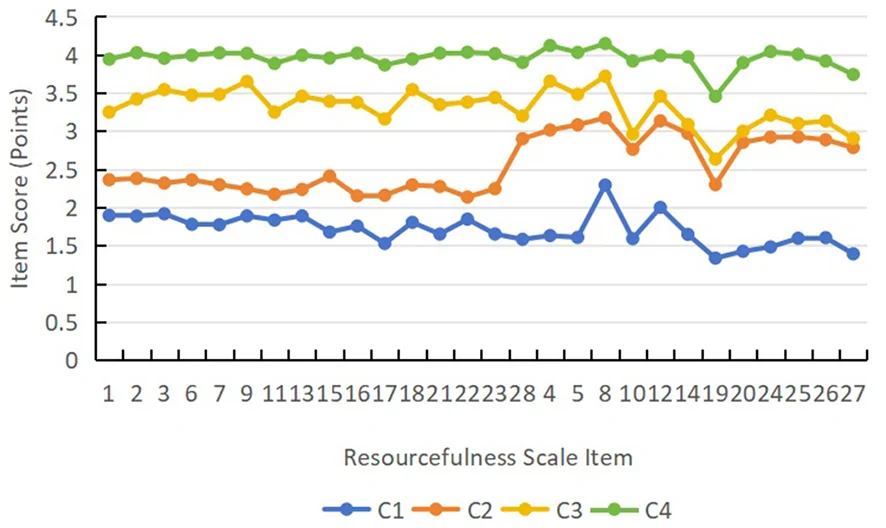
Distribution of resourcefulness latent profile characteristics in early postoperative patients with colorectal cancer ostomy. C1, low resourcefulness profile, C2, medium resourcefulness–intimate-reliant profile, C3, high resourcefulness–selectively help–seeking profile, C4, high resourcefulness profile.

Based on the latent profile analysis of scores from the Resourcefulness Scale, we estimated models ranging from one to five classes to determine the optimal fit structure. The fit indices for each model are presented in Table 2: as the number of classes increased, the values of AIC, BIC, and aBIC continuously decreased; the entropy values for the 2-class, 3-class, and 4-class models were all above 0.80, indicating good classification accuracy; the LMR and BLRT tests showed that the 2-class, 3-class, and 4-class models were all significantly superior to the previous model (all *p* < 0.05), while the 5-class model showed a non-significant LMR test (*p* = 0.383), and one of its classes contained only 4.59% of participants, which was too small for meaningful interpretation. Therefore, based on statistical fit, model parsimony, and interpretability, the 4-class model was selected as the optimal solution. This model divided patients into four latent classes, all of which showed significant differences in both personal and social resourcefulness dimensions (all *p* < 0.001). Class 1 (C1, *n* = 137, 28.60%) scored the lowest on all items, particularly in personal resourcefulness (26.78 ± 6.90) and social resourcefulness (19.64 ± 4.92), and was thus named the “Low Resourcefulness Profile”; Class 2 (C2, *n* = 83, 17.33%) showed moderate levels in both personal resourcefulness (34.06 ± 4.09) and social resourcefulness (34.84 ± 2.77), with social resourcefulness scores significantly higher than those in Class 1, reflecting moderate overall resourcefulness and a strong tendency toward social engagement, and was named the “Medium Resourcefulness–Intimate-Reliant Profile”; Class 3 (C3, *n* = 173, 36.12%) scored highest in personal resourcefulness (51.16 ± 3.92) but significantly lower in social resourcefulness (38.29 ± 3.55) compared to Class 4, reflecting exceptional self-regulation ability alongside a more selective and internally driven approach to seeking social support. This profile was thus named the “High Resourcefulness–Selectively Help–Seeking Profile”; Class 4 (C4, *n* = 86, 17.95%) scored highest in both personal resourcefulness (59.70 ± 4.17) and social resourcefulness (47.35 ± 2.85), demonstrating comprehensive and well-balanced high-level resourcefulness, and was named the “High Resourcefulness Profile.” Table 3 presents the descriptive statistics for personal and social resourcefulness across these four profiles, all of which showed significant differences in both dimensions (all *p* < 0.001). The distinct patterns of these profiles, particularly the contrasting levels of personal and social resourcefulness between C2 and C3, are visually depicted in Figure 2. This cross–profile comparison suggests that C2 and C3 represent qualitatively distinct adaptation strategies rather than simply higher vs. lower resourcefulness along a single continuum.

**Table 2:** Model fit indices for the latent profile analysis of resourcefulness.

Number of Profiles	AIC	BIC	aBIC	Entropy	LMRT (*p*)	BLRT (*p*)	Class proportions (%)
1	41,933.002	42,166.617	41,988.880	–	–	–	100
2	34,977.192	35,331.787	35,062.006	0.983	< 0.001	< 0.001	44.68/55.32
3	33,609.750	34,085.324	33,723.501	0.983	< 0.001	< 0.001	28.60/18.16/53.04
**4**	**32,858.245**	**33,454.799**	**33,000.933**	**0.966**	**0.0007**	**< 0.001**	**28.60/17.33/36.12/17.95**
5	32,490.973	33,208.506	32,662.597	0.973	0.3832	< 0.001	4.59/24.22/17.12/36.12/17.95

The 4-profile solution (in bold) was selected as the optimal model based on statistical fit indices, parsimony, and interpretability. Percentages may not sum to 100% due to rounding.

AIC, akaike information criterion; BIC, Bayesian Information Criterion; aBIC, sample-size adjusted BIC; LMRT, Lo-Mendell-rubin adjusted likelihood ratio test; BLRT, bootstrapped likelihood ratio test.

**Table 3:** Descriptive statistics of resourcefulness dimensions across the four latent profiles.

Dimension	C1 (*n* = 137) M (SD)	C2 (*n* = 83) M (SD)	C3 (*n* = 173) M (SD)	C4 (*n* = 86) M (SD)	*F*	*p*
Personal Resourcefulness	26.78 (6.90)	34.06 (4.09)	51.16 (3.92)	59.70 (4.17)	1,038.05	< 0.001
Social Resourcefulness	19.64 (4.92)	34.84 (2.77)	38.29 (3.55)	47.35 (2.85)	1,093.49	< 0.001

C1, low resourcefulness profile, C2, medium resourcefulness–intimate-reliant profile, C3, high resourcefulness–selectively help–seeking profile, C4, high resourcefulness profile. M (SD), Mean (Standard Deviation). All inter-profile differences were statistically significant (*p* < 0.001).

### Associated factors of latent profile membership

Univariate tests (chi-square tests) of latent profile analysis revealed significant differences in the distribution of sociodemographic characteristics among different resourcefulness profiles, including gender (χ^2^ = 13.579, *p* = 0.004), educational level (χ^2^ = 206.353, *p* < 0.001), marital status (χ^2^ = 35.775, *p* < 0.001), per capita monthly household income (χ^2^ = 22.649, *p* = 0.001), and participation in ostomy support meetings (χ^2^ = 92.025, *p* < 0.001; Table 4).

**Table 4:** Univariate analysis of demographic and clinical variables across the four resourcefulness profiles.

Variable	Category	C1 (*n* = 137) *n* (%)	C2 (*n* = 83) *n* (%)	C3 (*n* = 173) *n* (%)	C4 (*n* = 86) *n* (%)	χ^2^	*p*-value
Gender	Male	99 (72.3)	59 (71.1)	118 (68.2)	43 (50.0)	13.579	0.004
	Female	38 (27.7)	24 (28.9)	55 (31.8)	43 (50.0)		
Educational	Junior high or below	119 (86.9)	68 (81.9)	46 (26.6)	10 (11.6)	206.353	**< 0.001**
attainment	High school/Technical	10 (7.3)	8 (9.7)	70 (40.5)	29 (33.7)		
	College or above	8 (5.8)	7 (8.4)	57 (32.9)	47 (54.7)		
Marital status	Married	71 (51.8)	55 (66.3)	111 (64.2)	78 (90.7)	35.775	**< 0.001**
	Unmarried/Divorced/ Widowed	66 (48.2)	28 (33.7)	62 (35.8)	8 (9.3)		
Per capita monthly income (CNY)	< 3,000	60 (43.8)	31 (37.3)	67 (38.7)	31 (36.0)	22.649	0.001
	3,000–4,999	63 (46.0)	39 (47.0)	56 (32.4)	30 (34.9)		
	≥5,000	14 (10.2)	13 (15.7)	50 (28.9)	25 (29.1)		
Ostomy support	No	108 (78.8)	59 (71.1)	100 (57.8)	14 (16.3)	92.025	**< 0.001**
meetings	Yes	29 (21.2)	24 (28.9)	73 (42.2)	72 (83.7)		

C1, low resourcefulness profile; C2, medium resourcefulness–intimate-reliant profile; C3, high resourcefulness–selectively help–seeking profile; C4, high resourcefulness profile. Only variables with significant differences (*p* < 0.05) in the univariate analysis are shown for brevity. Full table available upon request.

To illustrate the distributional patterns, we examined the proportions of key characteristics across the four profiles. Married status, higher education (≥college), and ostomy support meeting attendance demonstrated clear upward gradients from C1 to C4 (51.8% → 90.7%; 5.8% → 54.7%; 21.2% → 83.7%, respectively). Conversely, the proportion of male patients decreased from C1 to C4 (72.3% → 50.0%). Although the proportion of patients with income ≥5,000 CNY also showed an increasing trend (10.2% → 29.1%).

The multinomial logistic regression model showed good fit to the data (χ^2^ = 324.43, df = 21, *p* < 0.001; Cox and Snell *R*^2^ = 0.49; Nagelkerke *R*^2^ = 0.53). Assessment of multicollinearity revealed no significant issues: VIF values ranged from 1.054 to 1.11, all well below the threshold of 10. No sparse-cell problems were detected, with all expected frequencies exceeding five (Table 5). Educational level emerged as one of the strongest associated factors: compared to patients with a college education or above, those with a junior high school education or below were significantly less likely to belong to the High Resourcefulness Profile (C4) (OR = 0.02, *p* < 0.001) and more likely to fall into the Low Resourcefulness Profile (C1). Marital status also had a significant impact, with married patients being 7.33 times more likely to belong to the High Resourcefulness Profile (C4) than those who were unmarried/divorced/widowed (*p* < 0.001). In terms of economic status, patients with a per capita monthly household income below 5,000 CNY were more likely to be classified into the low or medium resourcefulness profiles. Additionally, participation in stoma care exchange meetings was a strong positive associated factor; non-participants were far less likely to belong to the High Resourcefulness Profile (C4) (OR = 0.07, *p* < 0.001). Gender also showed predictive power in some comparisons—for example, males were less likely than females to belong to the High Resourcefulness Profile.

**Table 5:** Multivariable multinomial logistic regression predicting resourcefulness profile membership.

Comparison	Associated factor	Category (vs. Reference)	β	SE	Wald χ^2^	*p*-value	OR (95% CI)
	Educational attainment (ref: college or above)	Junior high school or below	−0.30	0.54	0.30	0.59	0.74 (0.26–2.16)
		High school/Technical secondary school	0.07	0.71	0.01	0.92	1.07 (0.27–4.29)
	Marital status (ref: unmarried/divorced/widowed)	Married	0.61	0.30	4.21	**0.04**	1.84 (1.03–3.31)
	Per capita monthly household income (ref: ≥5,000 CNY)	< 3,000 CNY	−0.45	0.45	0.98	**0.32**	0.64 (0.26–1.55)
		3,000–4,999 CNY	−0.33	0.44	0.57	**0.45**	0.72 (0.30–1.70)
	Participation in ostomy support meetings (ref: yes)	No	−0.38	0.33	1.38	**0.24**	0.68 (0.36–1.29)
C3 vs. C1^a^	Gender (ref: female)	Male	−0.08	0.31	0.07	0.80	0.92 (0.50–1.7)
	Educational attainment (ref: college or above)	Junior high school or below	−2.73	0.43	41.41	< 0.001	0.07 (0.03–0.15)
		High school/Technical secondary school	0.16	0.52	0.09	0.76	1.17 (0.42–3.25)
	Marital status (ref: unmarried/divorced/widowed)	Married	0.41	0.29	1.99	**0.16**	1.51 (0.85–2.69)
	Per capita monthly household income (ref: ≥5,000 CNY)	< 3,000 CNY	−0.97	0.41	5.56	**0.02**	0.38 (0.17–0.85)
		3,000–4,999 CNY	−1.16	0.41	8.11	**0.00**	0.31 (0.14–0.70)
	Participation in ostomy support meetings (ref: yes)	No	−0.85	0.31	7.66	**0.01**	0.43 (0.23–0.78)
C4 vs.C1^a^	Gender (ref: female)	Male	−0.82	0.39	4.38	**0.04**	0.44 (0.21–0.95)
	Educational attainment (ref: college or above)	Junior high school or below	−3.82	0.55	48.50	< 0.001	0.02 (0.01–0.06)
		High school/Technical secondary school	−0.45	0.58	0.60	**0.44**	0.64 (0.21–1.99)
	Marital status (ref: unmarried/divorced/widowed)	Married	1.99	0.48	17.15	< 0.001	7.33 (2.86–18.81)
	Per Capita monthly household income (ref: ≥5,000 CNY)	< 3,000 CNY	−0.82	0.51	2.54	**0.11**	0.44 (0.16–1.21)
		3,000–4,999 CNY	−0.90	0.51	3.20	**0.07**	0.41 (0.15–1.09)
	Participation in ostomy support meetings (ref: yes)	No	−2.66	0.42	40.21	< 0.001	0.07 (0.03–0.16)
C3 vs.C2^a^	Gender (ref: female)	Male	0.08	0.33	0.06	0.81	1.09 (0.56–2.09)
	Educational attainment (ref: college or above)	Junior high school or below	−2.44	0.45	29.39	< 0.001	0.09 (0.04–0.21)
		High school/Technical secondary school	0.09	0.56	0.03	0.87	1.10 (0.37–3.25)
	Marital status (ref: unmarried/divorced/widowed)	Married	−0.20	0.33	0.37	0.54	0.82 (0.43–1.55)
	Per capita monthly household income (ref: ≥5,000 CNY)	< 3,000 CNY	−0.52	0.43	1.49	**0.22**	0.60 (0.26–1.37)
		3,000–4,999 CNY	−0.83	0.42	3.96	**0.05**	0.44 (0.19–0.99)
	Participation in ostomy support meetings (ref: yes)	No	−0.47	0.32	2.11	**0.15**	0.63 (0.33–1.180)
C4 vs.C2^a^	Gender (ref: female)	Male	−0.66	0.40	2.64	**0.10**	0.52 (0.24–1.15)
	Educational attainment (ref: college or above)	Junior high school or below	−3.52	0.56	39.69	< 0.001	0.03 (0.01–0.09)
		High school/Technical secondary school	−0.51	0.60	0.73	**0.39**	0.60 (0.19–1.94)
	Marital status (ref: unmarried/divorced/widowed)	Married	1.38	0.50	7.68	**0.01**	3.98 (1.50–10.56)
	Per capita monthly household income (ref: ≥5,000 CNY)	< 3,000 CNY	−0.37	0.52	0.51	**0.48**	0.69 (0.25–1.91)
		3,000–4,999 CNY	−0.57	0.51	1.28	**0.26**	0.56 (0.21–1.52)
Participation in ostomy support meetings (ref: yes)	No	−2.28	0.43	28.31	< 0.001	0.10 (0.04–0.24)
C4 vs.C3^a^	Gender (ref: female)	Male	−0.74	0.31	5.68	**0.02**	0.48 (0.26–0.88)
	Educational attainment (ref: college or above)	Junior high school or below	−1.08	0.43	6.21	**0.01**	0.34 (0.15–0.79)
		High school/Technical secondary school	−0.61	0.34	3.25	**0.07**	0.55 (0.28–1.06)
	Marital status (ref: unmarried/divorced/widowed)	Married	1.58	0.43	13.42	< 0.001	4.85 (2.08–11.27)
	Per capita monthly household income (ref: ≥5,000 CNY)	< 3,000 CNY	0.15	0.38	0.16	0.69	1.16 (0.56–2.43)
		3,000–4,999 CNY	0.26	0.38	0.45	0.50	1.29 (0.61–2.71)
	Participation in ostomy support meetings (ref: yes)	No	−1.81	0.35	27.45	< 0.001	0.16 (0.08–0.32)

a: reference category for each comparison is the first profile listed (e.g., for C2 vs. C1, C1 is the reference). C1, Low Resourcefulness profile, C2: medium resourcefulness–intimate-reliant profile, C3, high resourcefulness–selectively help–seeking profile, C4, high resourcefulness profile. OR, odds ratio; CI, confidence interval.

### Psychosocial characteristics across the identified resourcefulness profiles

The one-way ANOVA results demonstrated statistically significant differences across the four resourcefulness profiles for all measured psychosocial variables—perceived stress, stoma acceptance, and stigma—as well as their respective subscales (all *p* < 0.001; Table 6). *Post-hoc* comparisons revealed a consistent, dose-response-like gradient in psychosocial adjustment, strongly associated with the level of resourcefulness. The Low Resourcefulness Profile (C1) presented the most adverse profile, with markedly elevated scores for perceived stress (total: 42.49 ± 7.19), particularly in feelings of loss of control (22.04 ± 3.69) and tension (20.45 ± 4.25), alongside the highest levels of overall stigma (81.85 ± 9.04) and its components, such as social exclusion (30.37 ± 3.45). Concurrently, this profile reported the poorest stoma acceptance (22.57 ± 5.25). In stark contrast, the High Resourcefulness Profile (C4) exhibited the most adaptive outcomes, characterized by the lowest stress (14.04 ± 4.32) and stigma (38.52 ± 11.56) and the highest stoma acceptance (41.31 ± 4.00). The two intermediate profiles, Medium Resourcefulness-Intimate-Reliant (C2) and High Resourcefulness-Selectively Help–Seeking (C3), consistently occupied the middle ground. Their scores on all dimensions and subscales were sequentially ordered from C1 to C4, thereby establishing a clear linear trend: as resourcefulness increased from C1 to C4, psychosocial adaptation improved progressively, manifesting as lower perceived stress and stigma, and higher stoma acceptance.

**Table 6:** Psychosocial characteristics across the four identified resourcefulness profiles.

Variable	C1 (*n* = 137)	C2 (*n* = 83)	C3 (*n* = 173)	C4 (*n* = 86)	*F*	*p*	η^2^	95% CI for η^2^	*Post-hoc*
Perceived stress (PSS)									
Total score	42.49 ± 7.19	28.82 ± 5.21	23.74 ± 6.53	14.04 ± 4.32	424.08	**< 0.001**	**0.73**	**0.70–0.76**	C1^a^ > C2^b^ > C3^c^ > C4^d^
Loss of control	22.04 ± 3.69	16.43 ± 3.73	12.64 ± 3.94	7.92 ± 3.01	301.31	**< 0.001**	0.66	0.62–0.69	C1^a^ > C2^b^ > C3^c^ > C4^d^
Tension	20.45 ± 4.25	12.39 ± 4.22	11.10 ± 3.84	6.12 ± 3.70	256.23	**< 0.001**	0.62	0.58–0.66	C1^a^ > C2^b^ > C3^c^ > C4^d^
Stoma acceptance (SAQ)									
Total score	22.57 ± 5.25	30.27 ± 7.39	37.27 ± 5.12	41.31 ± 4.00	275.66	**< 0.001**	0.64	0.59–0.67	C1^a^ < C2^b^ < C3^c^ < C4^d^
Importance	8.18 ± 2.50	10.53 ± 2.71	12.80 ± 2.05	13.85 ± 1.70	152.62	**< 0.001**	0.49	0.44–0.54	C1^a^ < C2^b^ < C3^c^ < C4^d^
Autonomy & acceptance	9.04 ± 2.40	12.12 ± 3.16	15.30 ± 2.42	17.33 ± 2.16	247.38	**< 0.001**	0.61	0.57–0.65	C1^a^ < C2^b^ < C3^c^ < C4^d^
Perceived support	5.35 ± 1.43	7.61 ± 1.89	9.17 ± 1.46	10.14 ± 1.32	233.93	**< 0.001**	0.60	0.55–0.64	C1^a^ < C2^b^ < C3^c^ < C4^d^
Stigma (SIS)									
Total score	81.85 ± 9.04	67.10 ± 12.38	58.59 ± 14.83	38.52 ± 11.56	228.08	**< 0.001**	0.59	0.55–0.62	C1^a^ > C2^b^ > C3^c^ > C4^d^
Social exclusion	30.37 ± 3.45	24.80 ± 4.83	21.55 ± 5.56	14.49 ± 4.70	210.38	**< 0.001**	0.57	0.52–0.61	C1^a^ > C2^b^ > C3^c^ > C4^d^
Financial insecurity	10.20 ± 1.49	8.55 ± 1.63	7.51 ± 2.23	4.84 ± 1.75	154.21	**< 0.001**	0.49	0.44–0.54	C1^a^ > C2^b^ > C3^c^ > C4^d^
Internalized shame	16.96 ± 2.49	13.83 ± 2.73	12.35 ± 3.31	7.72 ± 2.73	186.55	**< 0.001**	0.54	0.49–0.58	C1^a^ > C2^b^ > C3^c^ > C4^d^
Social isolation	24.31 ± 2.95	19.92 ± 3.92	17.18 ± 4.47	11.48 ± 3.76	208.33	**< 0.001**	0.57	0.52–0.61	C1^a^ > C2^b^ > C3^c^ > C4^d^

Data are presented as Mean ± Standard Deviation. C1, low resourcefulness profile; C2, medium resourcefulness–intimate-reliant profile; C3, high resourcefulness–selectively help–seeking profile; C4, high resourcefulness profile. One-way ANOVA was used for all comparisons. Assumptions of normality (Shapiro–Wilk test) and homogeneity of variance (Levene's test) were assessed for all variables. Due to unequal group sizes and evidence of heteroscedasticity for some variables, Welch's ANOVA was used for all analyses, with Bonferroni *post-hoc* tests for pairwise comparisons. Effect sizes are reported as η^2^ with 95% confidence intervals. Different superscript letters (a, b, c, d) indicate statistically significant differences between profiles at *p* < 0.05. All comparisons are unadjusted for multiple testing. M ± SD, Mean ± Standard Deviation. PSS, perceived stress scale; SAQ, stoma acceptance questionnaire; SIS, social impact scale.

## Discussion

This study employed latent profile analysis to identify significant profile heterogeneity in the resourcefulness levels of colorectal cancer patients following ileostomy surgery, distinguishing four latent categories with distinct characteristics: the Low Resourcefulness Profile (C1, 28.60%), the Medium Resourcefulness–Intimate-Reliant Profile (C2, 17.33%), the High Resourcefulness–Selectively Help–Seeking Profile (C3, 36.12%), and the High Resourcefulness Profile (C4, 17.95%). Significant differences were observed in scores across the total resourcefulness scale and its two dimensions—personal resourcefulness and social resourcefulness—among these four categories (all *p* < 0.001). Their characteristic distributions showed no overlap, with C3 being the most prevalent category.

The formation of the C1 profile reflects a state of comprehensive vulnerability in both personal and social resources when patients face a major health crisis. This study found that this profile scored the lowest on both the personal and social resourcefulness dimensions assessed by the Resourcefulness Scale. When individuals confront compounded stressors such as cancer and ostomy surgery, pre-existing deficiencies in cognitive, economic, and social reserves significantly constrain their adaptation process (Tsukanov et al., 2025; Luan et al., 2024). Our data indicate that within the C1 profile, 86.86% had an education level of junior high school or below, 89.79% had a per capita monthly household income of < 5,000 CNY, and only 21.17% had participated in an ostomy support exchange meeting. These characteristics suggest that patients with lower educational attainment, limited household income, and non-attendance at support meetings were significantly associated with belonging to C1 relative to C3 and C4; however, the difference between C1 and C2 was not statistically significant, suggesting that this factor is particularly relevant for distinguishing the most severely resource-depleted patients. Lower education levels directly constrain the acquisition and comprehension of health information. As commonly observed in chronic disease populations, educational attainment is closely associated with self-management efficacy, forming a fundamental constraint on the development of personal resourcefulness (Liang et al., 2024). Concurrently, financial strain not only exacerbates psychological burden but also substantially diminishes patients' capacity to engage in supportive activities and access quality care resources, thereby impairing the construction and utilization of their social support networks and resulting in a deficit in social resourcefulness (Ji et al., 2024). The convergence of these multidimensional resource disadvantages is directly manifested in this profile, which exhibits the highest levels of perceived stress, the strongest sense of stigma, and the lowest acceptance of ostomy. This aligns with findings by Guan et al. (2023) on the relationship between stress and resource scarcity. The pronounced stigma identified in our study also resonates with conclusions by Karaçar et al., which indicate that socioeconomic marginalization intensifies patients' experience of shame (Karaçar and Bademli, 2022). Furthermore, the markedly low ostomy acceptance corroborates the perspective of Lu et al., positing that psychological acceptance of an ostomy is severely impeded when patients are overwhelmed by stress and stigma (Lu and Jin, 2024).

The C2 profile appears to be characterized by a compensatory adaptation pattern. These patients heavily rely on intimate relationships. They also have limited personal coping resources. The label “intimate-reliant” captures this pattern. Their social resourcefulness is elevated. However, it seems to derive mainly from private, familiar networks. It does not come from broader professional or peer support. This is also suggested by their notably lower participation in formal support activities. This pattern is consistent with dyadic coping theory (Bodenmann, 1997). That theory elaborates how couples jointly appraise stressors. They also engage in collaborative coping (Wang et al., 2024). This aligns with (Gram et al., 2021), who identified marriage as a key protective factor in chronic illness. For married patients in the C2 group, the spouse appears to play a prominent compensatory role. The spouse may offset the patient's limited self-regulation capacity. However, their low utilization of formal professional support points to a considerable constraint. They may prefer familiar informal networks. Alternatively, they may actively avoid professional settings. Those settings require disclosing intimate disease details. They also require interacting with unfamiliar peers. (Wang et al., 2022) offer an explanatory framework. They suggest that anticipated discrimination or internalized shame may be associated with patients' tendency to “securitise” their support scope. Patients may confine support to the private sphere. The limitations of this adaptation pattern are reflected in their psychosocial outcomes. Their perceived stress (28.82 ± 5.21) and stigma (67.10 ± 12.38) were lower than those of C1. However, they remained substantially higher than those of C3 and C4. Their stoma acceptance (30.27 ± 7.39) showed only modest improvement. This observation is consistent with (Wei et al., 2025). They argued that social support must ultimately translate into internal cognitive restructuring and emotional acceptance. That would facilitate deep adaptation. This transition appears incomplete in this intimacy-reliant profile.

The formation of the C3 profile reveals an adaptive strategy centered on high self-reliance and maintaining a sense of control. This profile presents a distinctive pattern: exceptionally high personal resourcefulness (54.36) alongside upper-intermediate social resourcefulness (38.29). Although this social resourcefulness score is substantially higher than C1 (19.64) and C2 (34.84), it remains moderately lower than C4 (47.35). This pattern suggests that some patients prioritize self-regulation and independent problem-solving to preserve autonomy. From a behavioral perspective, this can be understood through Bandura's self-efficacy theory (Bandura, 1977). This theory posits that beliefs in one's capabilities influence effort, persistence, and emotional responses. Higher education may be associated with stronger efficacy beliefs regarding disease management. C3 patients have exceptionally high personal resourcefulness, and their educational level is relatively high (32.9% with college education or above). This heightened efficacy may manifest as a preference for autonomous coping: they rely primarily on internal capacities and seek help selectively. This does not mean they lack support—their social resourcefulness is upper-intermediate—but that they trust their own ability to manage most challenges independently (Hu et al., 2025). This is consistent with (Zhou et al., 2020), who found that higher educational attainment facilitates superior self-regulation strategies. These strategies enable patients to use cognitive approaches to maintain self-mastery (Rousset et al., 2025). However, their help-seeking is more selective and internally driven than C4, reflecting a preference for self-reliance unless external support is clearly necessary. Thus, the core characteristic defining this profile is the co-existence of “high personal resourcefulness” and “selective help-seeking.” This strategy appears adaptive yet may carry limitations. Selectivity may be associated with elevated stigma relative to C4 and an autonomy orientation. Notably, higher stigma may lead to anticipation of negative evaluation and proactive reduction of social exposure, consistent with (Mi et al., 2025). While personal resourcefulness appears to buffer distress—as reflected in lower psychological distress than C1 and C2—this strategy may reduce opportunities for cognitive restructuring and emotional validation through social interaction. This pattern may help account for lower stoma acceptance than C4, as stoma acceptance is closely tied to social integration (Li, X. et al., 2025).

The formation of the C4 profile represents an ideal state where intrinsic personal resources and external social resources interact positively to achieve synergistic enhancement. This profile demonstrates excellent and balanced levels in both personal and social resourcefulness dimensions. This study found that multiple protective factors jointly contribute to this optimal profile: a higher education level provides a foundation for cognitive strategies; marital status offers a stable system of emotional and instrumental support; and the most critical behavioral factor—active participation in ostomy support exchange meetings (with a participation rate of 83.7%)—constitutes a crucial “social learning platform.” This platform not only provides instrumental support (e.g., care techniques) that is positively associated with personal resourcefulness but, more importantly, serves as a safe environment. Within this context, receiving empathy and validation (Liang et al., 2024) correlates with stronger social resourcefulness, and peer observation appears to be related to the refinement of one's own strategies. In this sample, patients with higher education levels, who are married and actively engaged in professional support activities, tend to exhibit characteristics most closely aligned with this profile. However, given the cross–sectional design of this study, the observed association between support meeting attendance and resourcefulness should not be interpreted causally. It remains equally plausible that patients with higher resourcefulness were more inclined to attend such meetings, rather than attendance itself enhancing resourcefulness. This advantageous combination of resources translates directly into the best psychosocial adaptation outcomes: the C4 profile exhibited the lowest levels of perceived stress (14.04 ± 4.32), the lowest stigma (38.52 ± 11.56), and the highest stoma acceptance (41.31 ± 4.00). These results are consistent with existing literature: individuals with high resourcefulness perceive significantly lower stress (Gao et al., 2023) and experience lower levels of stigma (Zhang et al., 2023), while high stoma acceptance is closely associated with better psychosocial integration (Zhang et al., 2022). Thus, a close association is formed between “high and balanced resourcefulness” and “optimal adaptation outcomes” within this profile.

## Clinical implications

Nurses should assess resourcefulness profiles to guide tailored interventions. For C1, interventions should focus on comprehensive resource reinforcement, including structured health education and facilitated access to financial and social support. For C2, enhancing the quality of existing intimate support networks and safely guiding them toward diverse professional support sources is crucial. For C3, a “respecting autonomy while providing gentle guidance” approach is recommended, such as offering self-directed educational materials and creating low-pressure observational learning opportunities (e.g., attending support meetings as listeners). For C4, empowerment and maintenance through peer support roles can be beneficial. Second, actively encouraging participation in structured ostomy support meetings is a key modifiable factor associated with higher resourcefulness, and nurses should proactively facilitate this engagement. Additionally, encouraging participation in ostomy support meetings may be a promising strategy for enhancing patient resourcefulness, though this recommendation is hypothesis-generating and should not be interpreted as a confirmed intervention effect. Future longitudinal or intervention studies are needed to establish causality and determine the optimal frequency and format of such support.

## Study limitations

This study also has several limitations. First, the cross-sectional design precludes inferring causal relationships between potential profiles of resourcefulness and psychosocial adaptation variables (such as stress and acceptance). It remains unclear whether specific combinations of resourceful strategies led to better adaptation or whether better initial adaptation facilitated the development of certain resources. Additionally, the secondary analyses treated class membership as fixed without accounting for classification error, though classification quality was high. Future research using bias–adjusted methods (e.g., BCH) would strengthen validity. Second, the sample originated from tertiary hospitals in two central cities in western China. Although the sample size was adequate, the convenience sampling method may limit the generalizability of findings to patients across broader geographic regions or different levels of healthcare institutions. Third, the assessment of ostomy support meeting attendance was based on a single binary item (yes/no) without capturing the frequency, duration, or timing of participation relative to surgery. Finally, the study relied on self–reported data, which may be subject to social desirability bias, particularly regarding stigma. Patients may underreport stigma experiences due to shame or social judgment concerns, potentially attenuating observed associations.

## Conclusions

Resourcefulness among patients with colorectal cancer and an intestinal ostomy is heterogeneous, with four distinct profiles identified: Low Resourcefulness (C1), Medium Resourcefulness-Intimate-Reliant (C2), High Resourcefulness-Selectively Help–Seeking (C3), and High Resourcefulness (C4). Sociodemographic factors such as being married, having a higher education (college or above), and—most importantly—actively participating in ostomy support meetings were key factors distinguishing the C4 from the C1. Moreover, the proportions of patients with these factors, along with higher income, exhibited a clear progressive increase from C1 to C4. A clear, dose-response gradient exists from the low to high resourcefulness profiles, where higher and more balanced resourcefulness is associated with lower perceived stress, lower stigma, and greater stoma acceptance. These findings underscore the importance of a personalized, profile-based approach to psychosocial interventions for this population.

## Data Availability

The datasets presented in this article are not readily available because restrictions apply to the availability of these data, which were used under a data-sharing agreement in the present study and are therefore not publicly available. The data are, however, available upon reasonable request from the corresponding author. Requests to access the datasets should be directed to Hong Wu, 1044328830@qq.com.
